# Phenotypic Characterization of Autoreactive B Cells—Checkpoints of B Cell Tolerance in Patients with Systemic Lupus Erythematosus

**DOI:** 10.1371/journal.pone.0005776

**Published:** 2009-06-02

**Authors:** Annett M. Jacobi, Jie Zhang, Meggan Mackay, Cynthia Aranow, Betty Diamond

**Affiliations:** The Center for Autoimmune and Musculoskeletal Diseases, Feinstein Institute for Medical Research, North Shore-Long Island Jewish Health System, Manhasset, New York, United States of America; University of Miami, United States of America

## Abstract

DNA-reactive B cells play a central role in systemic lupus erythematosus (SLE); DNA antibodies precede clinical disease and in established disease correlate with renal inflammation and contribute to dendritic cell activation and high levels of type 1 interferon. A number of central and peripheral B cell tolerance mechanisms designed to control the survival, differentiation and activation of autoreactive B cells are thought to be disturbed in patients with SLE. The characterization of DNA-reactive B cells has, however, been limited by their low frequency in peripheral blood. Using a tetrameric configuration of a peptide mimetope of DNA bound by pathogenic anti-DNA antibodies, we can identify B cells producing potentially pathogenic DNA-reactive antibodies. We, therefore, characterized the maturation and differentiation states of peptide, (ds) double stranded DNA cross-reactive B cells in the peripheral blood of lupus patients and correlated these with clinical disease activity. Flow cytometric analysis demonstrated a significantly higher frequency of tetramer-binding B cells in SLE patients compared to healthy controls. We demonstrated the existence of a novel tolerance checkpoint at the transition of antigen-naïve to antigen-experienced. We further demonstrate that patients with moderately active disease have more autoreactive B cells in both the antigen-naïve and antigen-experienced compartments consistent with greater impairment in B cell tolerance in both early and late checkpoints in these patients than in patients with quiescent disease. This methodology enables us to gain insight into the development and fate of DNA-reactive B cells in individual patients with SLE and paves the way ultimately to permit better and more customized therapies.

## Introduction

A wide variety of autoantibodies can be detected in patients with systemic lupus erythematosus (SLE), a high percentage of which bind to nuclear antigens [1] . Autoantibodies to dsDNA are essentially diagnostic of the disease. They are considered to be pathogenic as changes in their titer correlate with disease activity, and in murine models they clearly contribute to lupus nephritis [2]. Furthermore, they help induce an “interferon signature” that results from activation of toll-like receptor (TLR) 9 in dendritic cells by DNA-containing immune complexes [3] Moreover, elevated titers of anti-DNA antibodies can be seen in patients before the onset of clinical disease [4]. The mechanisms underlying the failure to maintain tolerance that allow for maturation and activation of autoreactive cells in SLE that are specific for DNA remain incompletely understood. Murine models have demonstrated the existence of multiple tolerance checkpoints during B cell maturation and activation, both before and after the germinal center response [5], [6], [7], [8], [9]. Defects in many of these checkpoints have been reported in murine models of lupus; thus, each appears necessary to stave off pathogenic autoreactivity [10], [11], [12], [13].

A significant percentage of the immature B cell repertoire is autoreactive [14]. Negative selection of autoreactive B cells is mediated by at least 3 processes: receptor editing, anergy induction and deletion [15], [16], [17]. Autoreactive B cells that escape early tolerance induction may mature to become marginal zone or follicular cells depending on the nature of the interaction of the B cell receptor (BCR) with antigen and the local microenvironment. Thus, autoreactive B cells can exist as short-lived plasma cells or germinal center-matured memory cells and long-lived plasma cells [18], [19]. In murine lupus models, autoreactive B cells are phenotypically heterogeneous; genetic background, hormonal milieu and antigen exposure all contribute to this diversity [11], [18], [19], [20], [21], [22]. These observations predict the presence of extensive heterogeneity in patients with lupus.

Repertoire analysis of immature, transitional and naïve B cells of patients with SLE and non-autoimmune individuals has confirmed the presence of multiple tolerance checkpoints [14], [23], [24], [25]. An assessment of the percentage of self- or poly-reactive B cells in early B cell populations has revealed two tolerance checkpoints, one at the immature to transitional junction and another one at the transitional to naïve junction. In a study of a small number of lupus patients, it is clear that both of these tolerance checkpoints are incompletely maintained in SLE [25]. **Peripheral tolerance in patients with lupus also appears to be compromised.** Autoreactive B cells expressing a VH4-34 encoded Ig heavy chain and possessing the 9G4 idiotype are present in the mature B cell repertoire but are excluded from the germinal center in non-autoimmune individuals; they can, however, be readily found within tonsillar germinal centers in SLE patients [26].

Our laboratory previously identified a peptide sequence (DWEYS) that behaves as a dsDNA mimetope [27]. Antibodies binding this sequence can cause renal disease and brain disease in mice, and are detected frequently in serum of patients with SLE and in cerebrospinal fluid of patients with neuropsychiatric manifestations of SLE [28], [29], [30], [31]. Immunization of BALB/c mice with an octameric form of this peptide (DWEYS-MAP) results in production of pathogenic IgG anti-dsDNA antibodies, glomerular immunoglobulin deposition, proteinuria as well as excitotoxic neuronal loss following a breach in the blood-brain barrier [28], [29], [32].

A fluorochrome–labeled tetrameric DWEYS peptide (DWEYS-tetramer), with a higher avidity for peptide reactive B cells than monomeric peptide [33] was generated to identify the peptide/dsDNA-cross-reactive B cell population in the murine immune response. Using this reagent, we have previously identified peptide dsDNA-cross-reactive B cells in mice immunized with DWEYS-MAP [34], [35]. Furthermore, we have shown that B cells in the peripheral blood of lupus patients that bind the DWEYS-tetramer are highly enriched for peptide and DNA reactivity [33].

This reagent therefore enables us to track the development and fate of a subset of dsDNA-reactive B cells in individual patients with SLE and gain insight into the heterogeneity of the anti-DNA response. It additionally may facilitate the identification of clinical subsets that may differ with respect to disease phenotype and disease activity and in response to therapy. Our goal in this cross-sectional study was to use the tetrameric DWEYS peptide to evaluate the frequency of these autoreactive B cells in antigen-naïve and antigen-experienced B cell subsets in SLE patients.

## Methods

### Phenotypic characterization of tetramer-binding B cells by flow cytometry

#### Subject Population

Peripheral blood was drawn in tubes containing citrate from 22 SLE patients fulfilling the revised ACR criteria for SLE who attended the outpatient Rheumatology clinics at Jacobi, Montefiore and Columbia University Medical Centers. The protocol was approved by the Institutional Review Boards at all three institutions. Informed consent was obtained from all patients prior to enrollment. For study entry, all patients were required to be 18 years of age or older. Patients were excluded if they had known infection with hepatitis B, hepatitis C or HIV. Patients were assessed for clinical disease activity using the Systemic Lupus Erythematosus Disease Activity Index **(SLEDAI). Peripheral blood from a control group consisting of 10 healthy subjects (4 Caucasian, 4 Asian, 1 African-American and 1 Hispanic) was also analyzed.** Peripheral blood from a control group consisting of 10 healthy subjects was also analyzed. Peripheral blood mononuclear cells (PBMCs) were prepared for flow cytometric analysis using Ficoll Paque (GE Healthcare, Piscataway, NJ) density gradient centrifugation and plasma of all individuals was stored at −20°C for assessment of anti-dsDNA and anti-DWEYS peptide antibody levels.

DWEYSVWLSN-streptavidin-allophycocyanin tetramers were generated incubating 25 μl biotinylated peptide (650 μM) (AnaSpec, San Jose, CA) with 75 μl allophycocyanin-labeled streptavidin (6.1 μM) (Molecular Probes, Eugene, OR) at 4°C overnight. Subsequently, peptide–APC complexes were separated from free peptide by gel filtration using a Bio-Gel P-30 spin column (Bio Rad, Hercules, CA).

Immunofluorescence labeling for multicolor flow cytometric analysis was performed by incubating PBMCs with anti-human monoclonal antibodies to: CD3/CD14/CD16 (Pacific Blue, UCHT-1/M5E2/3G8), CD19 (PerCP, SJ25C1), IgD (FITC, IA6-2) (BD PharMingen, San Diego, CA), CD27 (PE, CBL27/1), CD10 (FITC-labeled SJ5-1B4) (Invitrogen, Carlsbad, CA). In addition, the tetramer was used to label DWEYS-peptide-specific B lymphocytes. Labeling was performed in PBS/0.5%BSA/5 mM EDTA at 4°C for 30 minutes. DAPI (4′,6-diamidino-2-phenylindole dihydrochloride, Molecular Probes) was added before flow cytometric analysis (220 nM) to identify dead cells. Flow cytometric analysis was performed using the LSRII (Becton Dickinson, San Jose, CA) and FlowJo software (Treestar Inc, Ashland, OR). Doublets were excluded from analysis. Up to 2.5×10^6^ events were acquired per analysis.

#### ELISAs

The ELISA assay for dsDNA-binding was performed as described [29]. Briefly calf thymus dsDNA, 25 μl per well at 100 μg/ml, was adsorbed to 96-well half area plates (Corning Life Science, Pittsburgh, PA), dried overnight at 37°C. DWEYS-peptide at 20 μg/ml in PBS was adsorbed to Immulon 2HB ELISA-plates (Thermo Electron Corporation, Waltham, MA) plates at 4°C overnight. The following day, plates were blocked with 2% BSA in PBS (dsDNA ELISA) or 3% FBS in PBS (peptide ELISA) for 1 h at 37°C. Plasma was diluted 1:300 (dsDNA ELISA) or 1:100 (peptide ELISA) in PBS before incubating plates for 1 h at 37°C. All ELISAs were developed with alkaline phosphatase conjugated goat anti-human IgG (Southern Biotechnology) and OD405 was measured using a Victor microplate reader (Perkin Elmer, Waltham, MA).

#### Statistical analysis

Frequencies of B cell subpopulations were calculated using FlowJo software (TreeStar). Differences in frequencies of tetramer-binding B cells between SLE patients and healthy controls and in different B cell subsets between patients grouped according to disease activity were determined using the unpaired, t tests. The Spearman's rank test was used to analyze if there was a correlation between the IgG anti-dsDNA-antibody and anti-DWEYS-antibody levels and the Mann Whitney U test to compare serological results of patients and normal individuals. The paired or unpaired t test **as well as ANOVA** were used to compare frequencies of tetramer-binding B cells within different B cell subsets. P values<0.05 were considered to be statistically significant. Data was analyzed using the GraphPad Prism4- (GraphPad, San Diego, CA).

## Results

### Tetramer-binding B cells in peripheral blood of lupus patients

**Figure 1:**
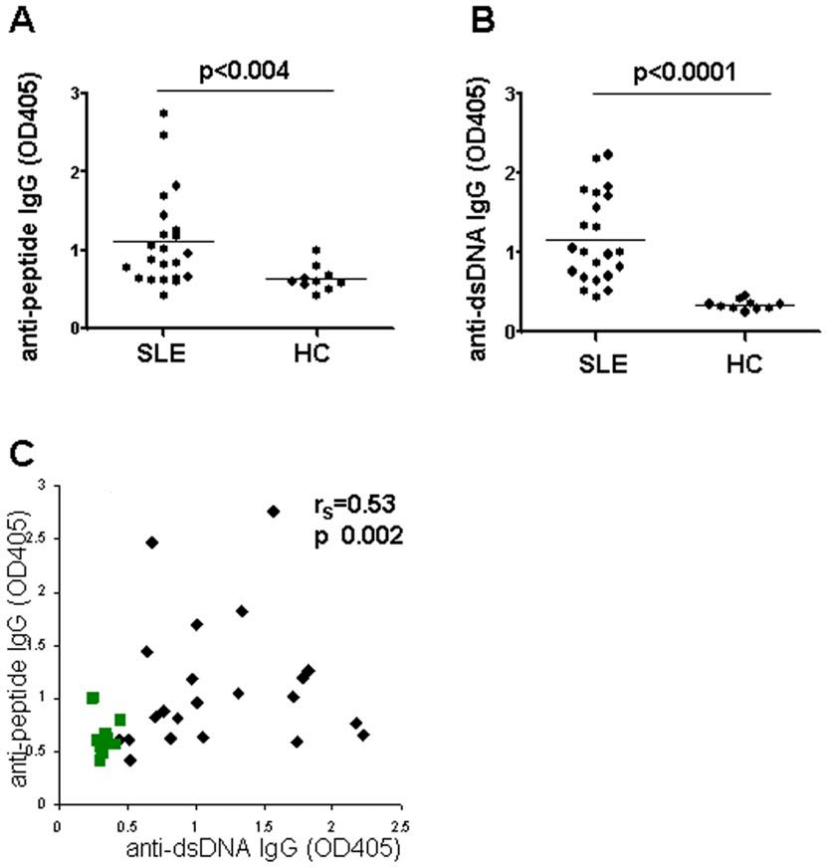
Antibody titters. A significant difference exists between the IgG anti-peptide (A) and anti-dsDNA (B) antibody levels in plasma samples of patients with SLE and healthy controls (Mann Whitney U-test, mean values are depicted). C. A significant correlation exists between anti-peptide IgG and anti-dsDNA IgG levels in all individuals analyzed. Healthy controls are shown in green, lupus patients in black (Spearman's rank correlation test).

To determine the frequency **of tetramer-binding B cells, peripheral blood cells of 22** patients with SLE (20 female, 2 male, 35.8±10.7 years old) and 10 healthy donors (8 female, 2 male, 32.2±7.9 years old) were analyzed by flow cytometry. The characteristics of the patient cohort are shown in Table 1. Both IgG anti-peptide antibody levels and IgG anti-dsDNA antibody levels determined by ELISA were significantly higher in SLE patients compared to healthy subjects (p<0.004, Figure 1A and p<0.0001, Figure 1B). Using routine clinical assays, 15 patients had elevated levels of anti-dsDNA antibodies (Table 1). Eleven patients had elevated anti-peptide antibody levels (at least 2SD above the mean of controls, Figure 1A). When all subjects were analyzed, a significant correlation between IgG anti-dsDNA and anti-peptide antibody levels was identified, (r_s_ = 0.53, p = 0.002) although some patients exhibited a discordance between anti-DNA and anti-peptide reactivity.

**Table 1:** Patient characteristics

disease manifestations									therapeutic regimen						ELISA
	age (years)	Gender	Ethnicity	SLEDAI	mucocutaneous	arthritis	nephritis#	decreased complement	Prednisone (mg)	HCQ*	methotrexate	azathioprine	MMP†	cyclophosphamide	IgG anti-dsDNA-antibody level
**J4**	42	F	A	2	x					x					n
**J6**	31	F	H	4	x			x		x			x		n
**J9**	34	M	A	2						x			x		p
**J24**	40	F	H	4				x		x					p
**C36**	39	F	H	0					5					x	n
**J1**	23	F	H	16	x	x	x	x	80				x		p
**J21**	41	F	A	2				n.d.	15	x					p
**J34**	44	F	A	4				x		x	x				p
**J50**	24	F	H	6	x			x		x			x		p
**J57**	22	F	H	12	x	x		x		x		x			p
**J59**	23	F	H	20	x		x	x	5						p
**C34**	42	M	H	2					20	x					p
**C35**	29	F	H	4	x			n.d.	20	x		x			p
**J7**	32	F	A	0					5				x		n
**J52**	35	F	A	1						x		x			n
**J60**	57	F	A	16			x	x	20				x		p
**J62**	29	F	A	4				x				x			p
**J63**	43	F	A	4		x			20						n
**C14**	22	F	H	8	x			x	15					x	p
**C30**	33	F	H	2					2.5				x		p
**C31**	41	F	A	6	x			x	25	x		x			p
**C42**	62	F	A	2				x							n

* HCQ = hydroxychloroquin.

† MMP = mycophenolate Mofetil.

n.d. = not determined.

# active nephritis (nephritic sediment or proteinuria (>0.5 g/day).

A = African-American,H = Hispanic.

**Figure 2:**
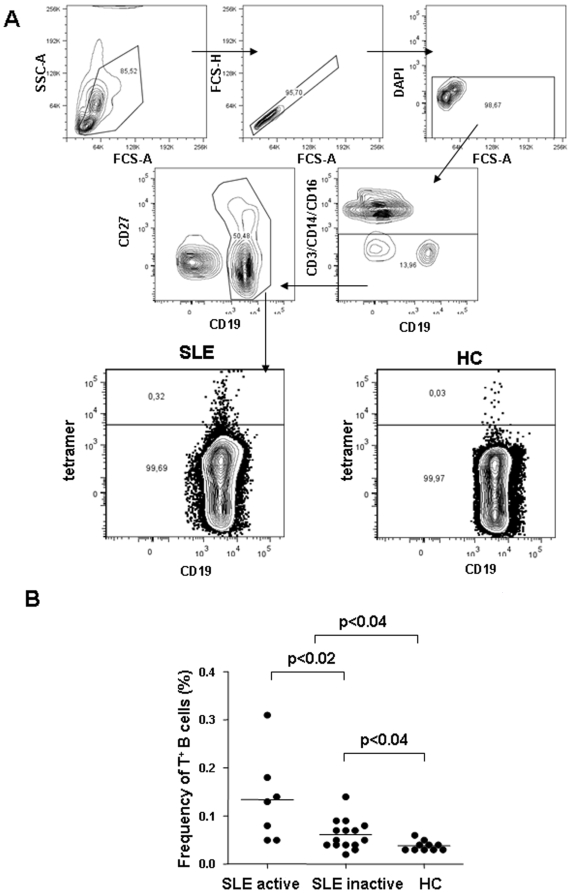
Tetramer-binding B cells. A: Gating strategy. PBMCs were incubated with monoclonal antibodies to human: CD3/CD14/CD16 and CD19. In addition, the tetramer was used to label DWEYS-peptide-specific B lymphocytes. DAPI was added before flow cytometric analysis to identify dead cells. Doublets were excluded from analysis. After gating out dead cells, doublets and CD3, CD14- or CD16-positive cells, B cells (CD19-positive cells) were gated and the frequency of tetramer-binding B cells was determined as shown in a patient with SLE and a healthy control (HC). B: Patients with SLE exhibit a significantly higher frequency of tetramer-binding B cells compared to healthy controls and active patients a significantly higher frequency of tetramer-binding B cells than inactive patients. (unpaired t test. Mean values are depicted). HC = healthy controls

Flow cytometric analysis of peripheral blood cells performed concurrently using the gating strategy shown in Figure 2A **demonstrated that all groups differed significantly (one way ANOVA p<0.002)** a higher frequency of tetramer-binding B cells **was present** in SLE patients compared to healthy donors (0.085±0.065% vs. 0.038±0.010%, p<0.04, Figure 2B). Patients with moderately active disease (SLEDAI>4) had a significantly higher frequency of tetramer-binding B cells than patients with quiescent disease (SLEDAI≤4) (0.134±0.091% *vs.* 0.061±0.031%, p<0.02). However, even patients with inactive disease had a significantly elevated frequency of tetramer-binding B cells compared to healthy donors (p<0.04).

### Phenotype of tetramer-binding B cells in the peripheral blood of lupus patients

**Figure 3:**
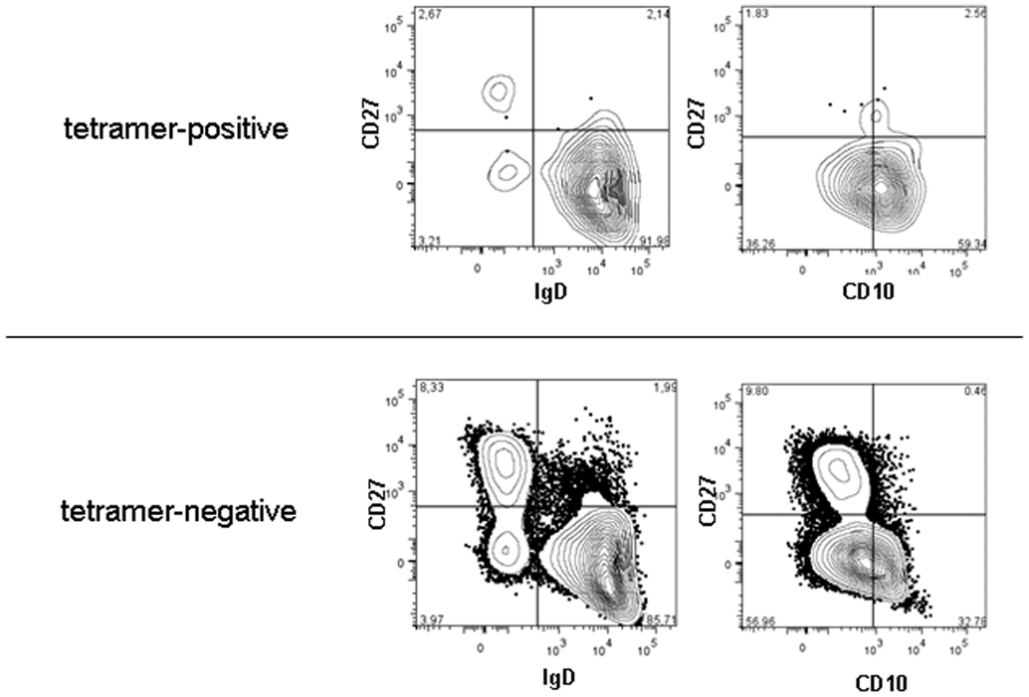
Gating strategy used to characterize tetramer-binding B cells further. In addition to the gating procedure depicted in Figure 2A, tetramer-binding and tetramer-negative B cells were subdivided in CD27^+^ memory B cells and CD27^++^ plasmablasts (antigen-experienced) and CD27^−^IgD^+^ antigen-inexperienced B cells, comprising both transitional (CD10^+^) and naïve (CD10^−^) B cells.

We are particularly interested in the regulation of B cells that have encountered antigen, as the checkpoints that are operative after antigen activation are less well studied. We, therefore, performed a detailed analysis of B cell subsets to determine the frequency of tetramer-binding B cells in antigen-inexperienced (CD27^−^IgD^+^), or antigen-experienced **subsets** including IgD^+^CD27^+^ and Ig-class-switched (IgD^−^CD27^+^) memory cell subsets and plasmablasts (CD27^++^CD19^low^) in individual patients with SLE. Since the CD27^−^IgD^−^ cell subset, which appears to be enhanced in the peripheral blood of patients with SLE [36], [37] might include B cells which may have undergone class switching simply by exposure to TLR-ligands or cytokines such as IL-10 or IL-21 or BAFF [38], [39], [40], [41], this subset was considered to be heterogeneous. It probably includes both, antigen-naive and antigen-experienced B cells, and was therefore excluded from final analyses. **This analysis was performed for SLE patients only as the frequencies were too low in control individuals to distinguish signal from background in most subpopulations.** The gating strategy for these analyses is depicted in Figure 3.

**Figure 4:**
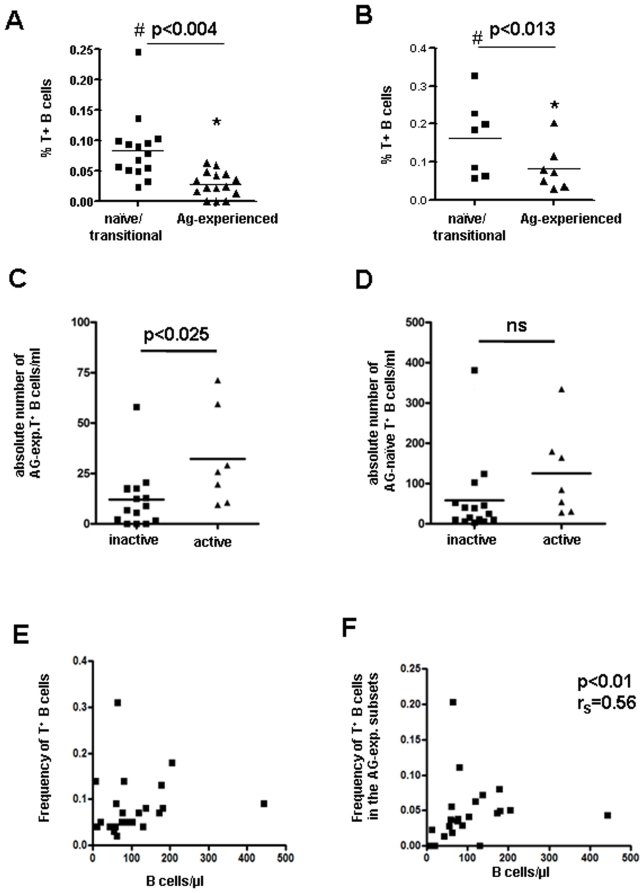
Late checkpoints of B cell tolerance. A significant difference exists between the frequency of tetramer-binding B cells within the antigen-inexperienced (naïve and transitional) B cell subsets and the antigen-experienced B cell subsets in patients with quiescent disease (A) and patients with active disease (B) (paired t test, mean values are depicted). The frequency of tetramer-binding cells was greater in both the antigen-naïve and the antigen-experienced subsets in patients with moderately active disease (n = 7) than in patients with quiescent disease (n = 15) (#p<0.025 and *p<0.005, respectively, unpaired t test). (C) The total number of tetramer-binding B cells in antigen-experienced subsets was greater in patients with active disease than in patients with quiescent disease (p<0.025, unpaired t test). (D) the, total number of tetramer-binding B cells in the antigen-naïve subsets was not different between the two patient groups. (E) The total B cell number and frequency of tetramer-binding B cells were not significantly correlated although there was a relationship between total B cell number and the frequency of tetramer-binding B cells in antigen-experienced subsets (r_S_ = 0.56, p<0.01, Spearman's rank correlation test) (F).

The frequency of tetramer-binding cells was greater in both the antigen-naïve and the antigen-experienced subsets in patients with moderately active disease (n = 7) than in patients with quiescent disease (n = 15) (p<0.025 and p<0.005, respectively) (Figure 4 **A and B)**. Interestingly, we observed a lower frequency of tetramer-binding B cells in the antigen-experienced population than in antigen-naïve population in both patients with quiescent disease and patients with moderately active disease (p<0.004 and p<0.013, respectively), demonstrating that there is a partial, although inadequate, maintenance of tolerance checkpoints after antigen activation even in active lupus patients (Figure 4 **A and B)**. Indeed, every patient exhibited a decrease in frequency of tetramer-binding B cells in the antigen-experienced population compared to the antigen-naïve population.

Interestingly, when we analyzed total number of tetramer-binding B cells with an antigen-experienced phenotype we found that patients with active disease had significantly higher numbers compared to patients with quiescent disease (p<0.025). In contrast, we observed no difference in the total number of tetramer-binding naïve B cells in the two patient groups (Figure 4C and D). This analysis may, however, be distorted by one patient with inactive disease and high numbers of naïve tetramer-binding B cells. If this one patient was excluded this difference became statistically significant as well (p<0.02).

We next asked whether the frequency of tetramer-binding cells was related to total B cell number. We reasoned that B cell lymphopenia might lead to increased BAFF levels and impaired negative selection. Contrary to our expectation, there was no relationship between total B cell number and frequency of tetramer-binding B cells (Figure 4E), although there was a relationship between total B cell number and the frequency of tetramer-binding B cells in antigen-experienced subsets (r_S_ = 0.56, p<0.01,Figure 4F).

**To analyze** whether differences in ethnicity might associate with differences in B cell regulation Hispanic (n = 11) and African American (n = 11) patients **were compared.** No significant difference was observed between these ethnic groups with respect to frequency of tetramer-binding B cells within both the antigen-naive and the antigen-experienced subsets. With only 2 African-American patients with moderately active disease, it was not possible to ascertain the impact of ethnicity on the tolerance checkpoint at the antigen-to-antigen-naïve experienced junction.

**Figure 5:**
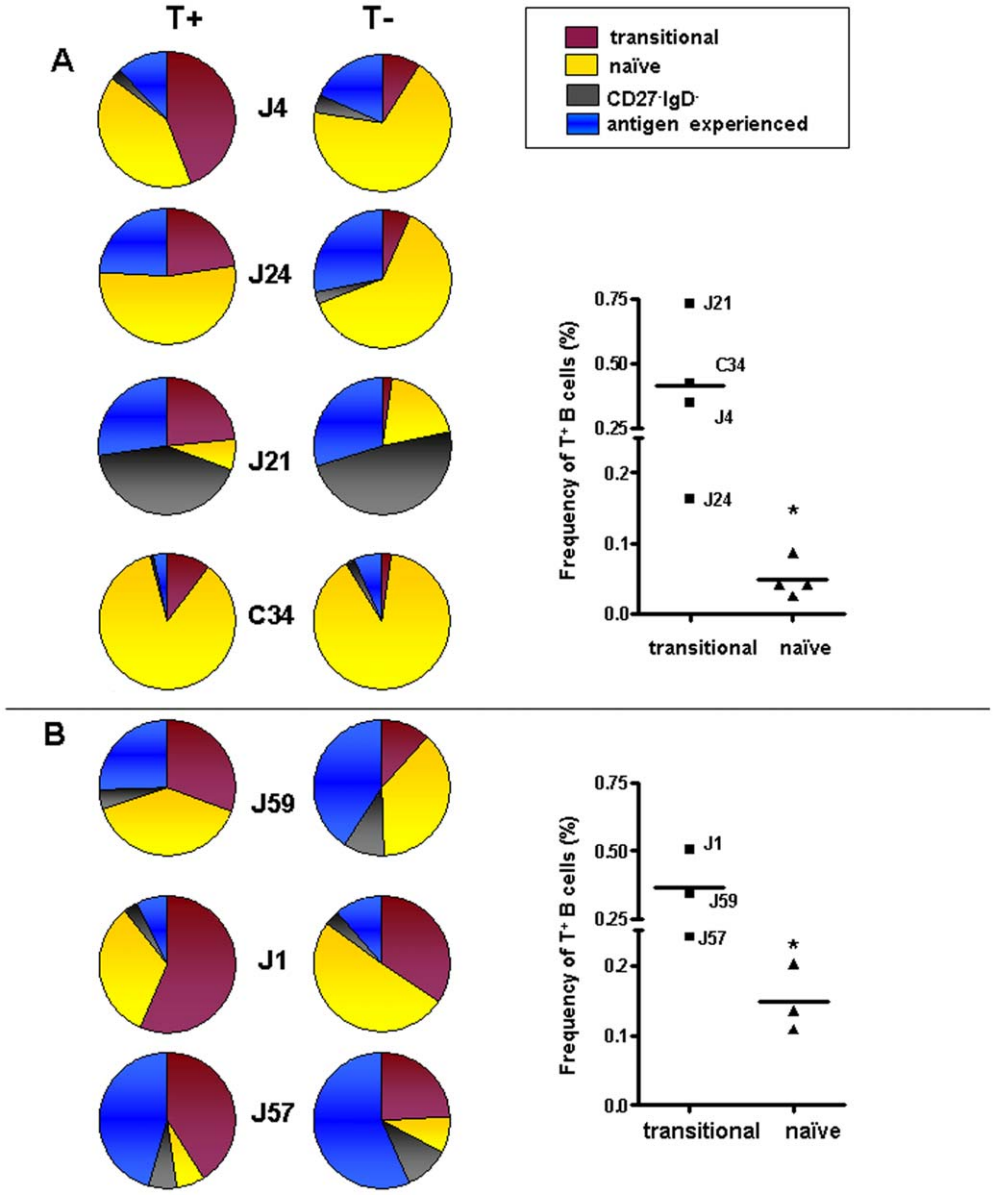
Early checkpoints of B cell tolerance. Phenotype of tetramer-binding B cells in 7 patients with SLE in patients with quiescent disease, defined as a SLEDAI≤4 (A) or patients showing signs of clinical disease activity, defined as a SLEDAI>4 (B). The percentage of tetramer-binding and tetramer-negative B cells with a transitional phenotype (bordeaux) and the calculated percentage of tetramer-binding (T+) and tetramer-negative (T-) B cells with a naïve phenotype (yellow) in 7 patients with SLE. The percentage of naïve B cells was calculated by subtracting the percentage of CD27^−^CD10^+^ B cells from the percentage of CD27^−^IgD^+^ B cells. The percentage of tetramer-binding and tetramer-negative B cells with an antigen-experienced phenotype comprising CD27^+^ switched (IgD^−^) and non-switched (IgD^+^) memory B cells and CD19^low^CD27^++^ plasmablasts is shown in blue. CD27^−^IgD^−^ B cells (grey) were excluded from analysis. The right panel of the figure shows the frequencies of tetramer-binding B cells within the transitional and naïve B cell subsets in these lupus patients. Mean values are shown. *Patients with moderately active disease exhibited a significantly higher frequency of tetramer-binding B cells in the naïve population (p<0.02, unpaired t test).

We also studied the transitional to naïve B cell checkpoint in a subset of patients (n = 4 with quiescent disease and n = 3 with moderately active disease). In each patient, there was again a reduction in tetramer-binding B cells as the B cells matured from the transitional compartment to the naïve compartment (Figure 5). Although the number of individuals was limited, these differences almost reached statistical significance for patients with quiescent disease **(p = 0.058)** and for patients with moderately active disease **(p = 0.05).** Interestingly, while the frequency of tetramer-binding B cells was comparable in the transitional population for both patients with quiescent disease and patients with moderately active disease (0.36±0.13% *vs.* 0.42±0.24%, respectively), patients with moderately active disease exhibited a significantly higher frequency of tetramer-binding B cells in the naïve population (0.15±0.05% *vs.* 0.05±0.03%, p<0.02), suggesting a greater impairment at this checkpoint in patients with moderately active disease.

## Discussion

The mechanisms underlying the failure of B cell tolerance as well as the contribution of different B cell subsets to the pathogenesis of SLE remain incompletely understood and the degree of heterogeneity in these parameters among lupus patients has not been extensively investigated. This study employed a tetrameric fluorochrome-labeled form of a dsDNA mimetope to characterize dsDNA-specific B cells obtained from the peripheral blood of patients with SLE. The tetramer was previously successfully used to track the peptide-specific B cell population in peptide-immunized BALB/c mice [33], [34]. Using the same tool, we were recently able to identify and enrich a rare peptide-specific B cell population in patients with SLE, and to demonstrate that the antibodies derived from these B cells bound peptide and were largely cross-reactive to dsDNA [35]. We now show that this cross-reactive tetrameric peptide can be used to investigate tolerance checkpoints in SLE patients. We noted that all lupus patients had a higher frequency of tetramer-binding B cells than did healthy controls.

A characterization of these tetramer-binding B cells confirmed the anticipated heterogeneity of patients with SLE. Most tetramer-binding B cells were antigen-naïve (70%), consistent with a previous study showing that lupus patients can have a high frequency of ANA-reactive B cells in the antigen-naïve B cell subsets even when in clinical remission or serologically inactive [25]. We identified a checkpoint for maintaining B cell tolerance that occurs during or after antigenic encounter. This checkpoint at the transition of autoreactive B cells from an antigen-naïve to an antigen-experienced compartment was present in lupus patients whether quiescent or moderately active. There was also a diminution of tetramer-binding B cells in lupus patients as B cells progressed from the transitional subset to the naïve subset although this was studied in a smaller number of patients. Interestingly, this checkpoint was more compromised in those patients with moderately active disease. It seems probable that multiple checkpoints are each partially compromised in lupus patients. Whether these checkpoints utilize similar or distinct mechanisms to tolerize autoreactive B cells, remains to be determined.

It has been reported that more active patients display high serum levels of BAFF [42]. We believe this may account for the greater impairment in the early transitional to naïve B cells checkpoint present in patients with moderately active disease. In mice, elevated BAFF levels permit the survival of autoreactive transitional B cells that would normally not mature to become naïve, immunocompetent B cells [43]. The increased BAFF levels in active patients may reflect increased BAFF production by dendritic cells exposed to nucleic acid-containing immune complexes, or could be related to disease-associated or therapy-associated B cell lymphopenia in both patients with active and patients with quiescent disease.

The decreased expression of FcγRIIb on memory B cells or immediate plasma cell precursors which occurs in approximately 50% of SLE patients may be one mechanism which contributes uniquely to the observed diminished tolerance in antigen-experienced B cells as FcγRIIb inhibits the B cell response in post-germinal center compartments and regulates plasma cell homeostasis [44], [45], [46], [47]. Classical mechanisms of B cell tolerance that have been demonstrated in the mouse such as follicular exclusion might also be impaired in patients with SLE due to lower FcγRIIb expression [48] or due to diminished competition from a non-autoreactive B cell population [49]. It is not clear if B cell lymphopenia, characterized by increased BAFF expression, affects late tolerance checkpoints as well.

It is necessary to note that patients in this study were on a variety of medications (Table 1). Whether these also contributed to aspects of the B cell repertoire cannot be assessed as the medication regimens in our patient cohort were too varied. Additional studies of patients selected for medication use will be needed to address this question.

Overall, lupus patients in this study displayed an increased frequency of autoreactive B cells in the early and late B cell repertoire independent of disease activity. Furthermore, an impairment of early selection checkpoints was associated with a greater disease activity and a significantly higher frequency of autoreactive B cells in the antigen-experienced B cell subsets. Given the phenotypic diversity of anti-DNA reactive B cells and the diverse tolerance mechanisms that are abrogated in murine models of SLE, the methodology we have used will be informative in longitudinal studies to track the changes in frequency and phenotype of autoreactive cells as individual patients progress from clinically quiescent disease to flare and back to quiescence. The methodology allows for an analysis of the representation of autoreactive B cells in different B cell subsets in a far larger number of patients than can be examining in methodologies that rely on cloning antibodies and expressing from individual B cells. Determining which mechanisms operate early in selection and which operate following antigen-activation, and which of these are impaired in SLE promises to be an important guide to identify immunologically distinct patient cohorts and to improve and customize therapeutic strategies.
